# Human Cytomegalovirus (HCMV) Shedding and T-cell Immune Responses in HCMV-seropositive Women During Pregnancy and Postpartum: Prevalence, Natural History, and Risk Factors

**DOI:** 10.1093/cid/ciag076

**Published:** 2026-02-16

**Authors:** Shari Sapuan, Ngee Keong Tan, David Carrington, Vanessa Greening, Christine E Jones, Asma Khalil, Cassie F Pope, Blair L Strang, Sarah White, Paul T Heath

**Affiliations:** Children's Services, St George's University Hospitals NHS Foundation Trust, London, United Kingdom; Centre for Neonatal and Paediatric Infection, School of Health and Medical Sciences, City St George's, University of London, London, United Kingdom; Department of Medical Microbiology, South West London Pathology, St George's University Hospitals NHS Foundation Trust, London, United Kingdom; School of Biological Sciences, Faculty of Biology, Medicine and Health, University of Manchester, Manchester, United Kingdom; Infection Care Group, St George's University Hospitals NHS Foundation Trust, London, United Kingdom; Centre for Neonatal and Paediatric Infection, School of Health and Medical Sciences, City St George's, University of London, London, United Kingdom; Faculty of Medicine and Institute for Life Sciences, University of Southampton, Southampton, United Kingdom; NIHR Southampton Clinical Research Facility and NIHR Southampton Biomedical Research Centre, University Hospital Southampton NHS Foundation Trust, Southampton, United Kingdom; Vascular Biology Research Centre, Molecular and Clinical Sciences Research Institute, City St George's University of London, London, United Kingdom; Fetal Medicine Unit, St George's Hospital, St George's University of London, London, United Kingdom; Infection Care Group, St George's University Hospitals NHS Foundation Trust, London, United Kingdom; Institute for Infection and Immunity, School of Health and Medical Sciences City St George's, University of London, London, United Kingdom; Institute for Infection and Immunity, School of Health and Medical Sciences, City St George's, University of London, London, United Kingdom; Department of Global, Public and Population Health and Policy, City St George's, University of London, London, United Kingdom; Centre for Neonatal and Paediatric Infection, School of Health and Medical Sciences, City St George's, University of London, London, United Kingdom

**Keywords:** cytomegalovirus, pregnancy, postpartum, shedding, seropositive

## Abstract

**Background:**

Human cytomegalovirus (HCMV) during pregnancy and poor immune control of HCMV are associated with adverse outcomes. Limited data exist on the prevalence, natural history, and risk factors of HCMV shedding and T-cell immune responses during pregnancy and postpartum in HCMV-seropositive women.

**Methods:**

Samples from 160 HCMV-seropositive women were collected at 3 time points during pregnancy and once postpartum. Shedding was determined by detecting HCMV DNA in saliva, urine, and vaginal secretions by quantitative polymerase chain reaction. HCMV-specific T-cell immune responses were determined by detecting interferon-gamma released in blood by QuantiFERON-CMV and T-SPOT.CMV assays. Information on demographics and contact with children's bodily fluids was collected.

**Results:**

The prevalence of HCMV shedding in HCMV-seropositive women in any bodily fluids was 18.8% [95% CI: 13.0–25.7%] during pregnancy and 21.3% [95% CI: 15.2–28.4%] including postpartum. Ethnicity [OR 0.2, 95% CI: 0.05–0.95, *P* = .043] and gravidity [OR 0.2, 95% CI: 0.05–0.94, *P* = .042] were associated with detection of shedding. Shedding quantity was associated with contact with children's saliva [Incidence rate ratio 1.98, 95% CI: 1.69–2.33, *P* < .001]. The prevalence of T-cell immune responses was ≤75% and almost 100% using QuantiFERON-CMV and T-SPOT.CMV, respectively. T-cell immune responses did not correlate with shedding.

**Conclusions:**

Around 1 in 5 HCMV-seropositive women shed HCMV during pregnancy and postpartum. Ethnicity and gravidity are associated with shedding, but not T-cell immune responses, and the quantity of shedding is associated with contact with saliva. Further studies investigating HCMV shedding, immune responses and their risk factors in women during pregnancy and postpartum are warranted.

## Teaser

Around 20% of HCMV-seropositive women were found to shed HCMV during pregnancy and postpartum. Ethnicity and gravidity, but not T-cell immune responses, were associated with shedding and the quantity of shedding was higher following exposure to children's saliva.

Congenital cytomegalovirus (cCMV) infection is the most common congenital infection worldwide [1]. Children with cCMV infection have life-long morbidity, including sensorineural hearing loss, neurodevelopmental disorders, and visual impairment [1–3].

cCMV can occur following primary or nonprimary human cytomegalovirus (HCMV) infection in pregnancy [4–6]. Both primary and nonprimary infections are associated with the excretion of HCMV in bodily fluids such as urogenital secretions, saliva, and breastmilk [7]. The detection of HCMV DNA in these bodily fluids by polymerase chain reaction (PCR) is termed shedding [1, 7].

To date, research on the prevention of acquisition and vertical transmission of HCMV has focused on HCMV-seronegative women, as primary infection has a greater risk of transmitting HCMV to the fetus [8–10]. In addition, primary infection in pregnancy can be diagnosed using serology, and maternal HCMV-seropositivity is associated with a degree of protection against vertical transmission [11, 12]. However, nonprimary infection in HCMV-seropositive pregnant women is a significant cause of cCMV and results in the majority of cCMV cases globally [11, 13]. Limited data exist on the prevalence, natural history, and risk factors of HCMV shedding during pregnancy and postpartum in HCMV-seropositive women. A greater understanding of HCMV shedding characteristics in these women is essential for the evaluation of preventative and therapeutic strategies, such as vaccine development and risk-reduction measures.

Effective immune control of HCMV is crucial to prevent HCMV disease and may be important in controlling HCMV infection, shedding, and vertical transmission [14–16]. While extensive studies have investigated host innate and adaptive (T cell and B cell) immune responses in primary infections in pregnant women, none have investigated HCMV-specific T-cell immune responses in nonprimary infections in seropositive pregnant women using commercially available interferon gamma (IFN-γ) release assays (IGRA) such as QuantiFERON-CMV or T-SPOT.CMV [14, 17–21]. Investigating the performance of these assays will not only improve the current understanding of T-cell functional control of HCMV during pregnancy, but also shed light on the potential utility of IFN-γ as a surrogate marker for maternal infection.

This study aimed to assess the prevalence, quantity and natural history of HCMV shedding and T-cell immune responses in HCMV-seropositive women during pregnancy and postpartum, as well as the risk factors for shedding.

## METHODS

### Study Design

A single-center longitudinal prospective cohort study entitled *Cytomegalovirus Shedding Characteristics in Pregnant Women* (cCHIPS) was conducted in an ethnically diverse population in London, United Kingdom (Clinicaltrials.gov identifier NCT04021628). Pregnant women attending their first antenatal appointment between March 2019 and December 2020 were approached for recruitment. Written informed consent was sought from those who met the study eligibility criteria: aged over 18 years, with sufficient understanding of English, living with at least 1 child aged <4 years, and with no immune deficiency. Women found to be HCMV-seropositive and with no serological evidence of acute infection (defined by detection of HCMV IgG and absence of HCMV IgM [Elecsys assays, Roche Diagnostics, US], and high HCMV IgG avidity [VIDAS, bioMerieux, France]) were recruited. The study was approved by the NHS Health Research Authority and London–Brent Research Ethics Committee (19/LO/0161).

Study visits occurred at 3 time points (T) during pregnancy (T1, 12–16 gestational weeks [GW]; T2, 17–26 GW; T3, 27 GW to predelivery) and at one time point during postpartum (T4, ≤6 weeks postdelivery). At each visit, self-sampling of saliva, midstream urine, and vaginal secretions using validated collection devices was performed [22, 23] (Supplementary Figure 1). Samples were transported at 15–25°C and stored at −80°C within 10 hours. A modified self-sampling instruction was provided during the COVID-19 pandemic, as study visits were not possible (Supplementary Figure 2). During this period, samples were stored in participants' home freezers at −20°C immediately following collection. Venepuncture was performed at all visits when consent was given. Participants also completed a demographic questionnaire (at first visit) and a hygiene-related contact questionnaire at all visits (Supplementary Figure 3).

### Quantitative PCR and T-cell Response Assays

Saliva, urine, and vaginal secretion samples were tested for HCMV DNA by quantitative PCR using the methodology and analysis described elsewhere [24]. The limit of detection for saliva, urine, and vaginal secretion samples was 200, 31, and 81 IU/ml, respectively. Blood samples were tested for IFN-γ released in plasma and culture media using a HCMV-specific QuantiFERON enzyme-linked immunosorbent assay (ELISA), QuantiFERON-CMV (QIAGEN GmbH, Germany) [25], and a HCMV-specific enzyme-linked immunospot (ELISPOT) assay, T-SPOT.CMV (Oxford Immunotec, UK), respectively, as per the manufacturer's protocol. The QuantiFERON-CMV assay consisted of a pool of HCMV-specific peptides, which selectively stimulated CD8+ T cells [25]. The T-SPOT.CMV assay consisted of separated HCMV-specific IE-1 and pp65 peptides, which stimulated effector cells purported to be CD4+ and CD8+ T cells [26]. The QuantiFERON-CMV assay quantitatively measured IFN-γ as international units, with a cutoff value of 0.2 IU/ml for positivity [25]. The T-SPOT.CMV assay quantitatively measures IFN-y producing T cells as spot-forming colonies produced per 250 000 peripheral blood mononuclear cells, where a greater number of spots indicated greater number of IFN-γ-producing cells, and the cutoff value for positivity was at least 1 spot count (either IE-1 or pp65) [26].

### Statistical Analysis

The sample size for the study was calculated based on a published estimate of shedding prevalence of 32% of pregnant women [27]. Assuming a population proportion of 30%, a sample size of 200 would have a 95% confidence level of 24–36%. Study data were collected and managed using Research Electronic Data Capture database [28] hosted at St George's, University of London. Descriptive statistics were generated on the number of women who were recruited and completed the study.

The prevalence and quantity of HCMV shedding and T-cell immune responses were analyzed using descriptive statistics. Prevalence was reported as frequency and percentage, with 95% confidence intervals (CIs) for percentages. The quantity of shedding and T-cell immune responses were summarized using the median, lower quartile (LQ), and upper quartile (UQ). Both variables were stratified by sample or assay type and time point. The agreement between the QuantiFERON-CMV and T-SPOT.CMV assays was analyzed using descriptive statistics.

Demographic (age, ethnicity, gravidity, birth and duration of living in the UK, education level, number and age of children, and work with children) and 4 hygiene-related factors were evaluated as risk factors associated with HCMV shedding detection (detected/not detected HCMV DNA) in any sample types and time points using simple logistic regression models. The 4 hygiene-related factors were derived from the frequency of contact with children's bodily fluids assessed in the hygiene-related contact questionnaire and extracted using principal component analysis that explores if the information it contains can be explained by fewer factors (Supplementary Figure 4). Differences in the 4 hygiene-related factors over time were assessed by one-way analysis of variance. To examine for risk factors associated with HCMV shedding prevalence, accounting for repeated measures by sample types and time points, mixed-effects logistic regression models were used, including a subject identifier variable as a random effect to account for within-subject correlations.

To analyze HCMV shedding quantity, mixed-effects zero-inflated negative binomial regression models were employed, with negative test results assigned a quantity of 0. This method accounted for overdispersion and excess zeros in the data. Covariates of interest were also assessed for their influence on shedding quantity.

To evaluate variation in HCMV shedding prevalence and quantity across sample types and time points, univariate associations of sample type and time point with the dependent variables were initially tested using the described regression methods. Multivariable models were then fitted, simultaneously, including sample type and time point. Interaction terms between sample type and time point were explored to assess effect modification; however, these models failed to converge and were excluded from the final analysis.

To assess the association of T-cell immune responses (quantitative results of QuantiFERON-CMV and T-SPOT.CMV assays) with HCMV shedding detection, accounting for repeated measures by time points, mixed-effects logistic regression was used.

The mixed-model approach ensured that all collected data were utilized. Missing data were assumed to be missing at random. Risk factors with a univariate association at a significance level of <0.1 were included in multivariable models. A 5% significance level was applied for all other analyses. All regression models were implemented in RStudio, and additional analyses were conducted using IBM SPSS Statistics (version 29).

## RESULTS

### Study Population

**Figure 1.**
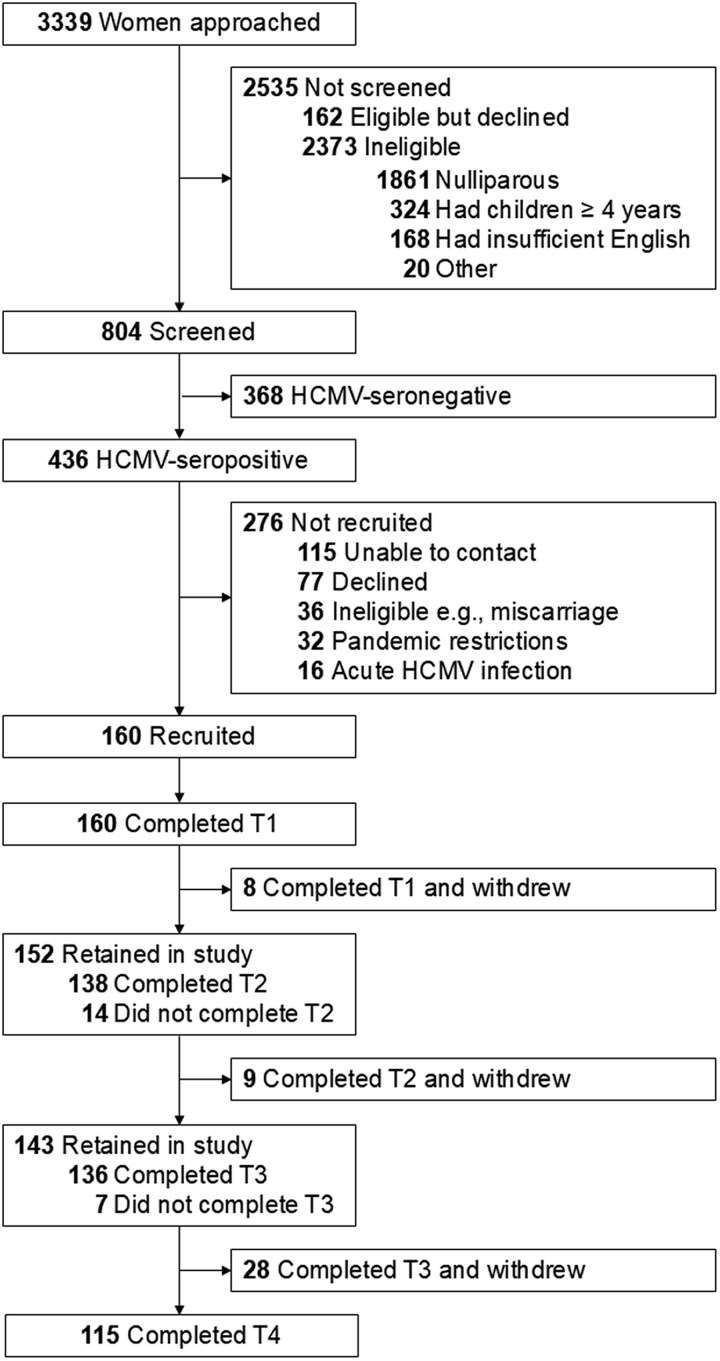
Recruitment and follow-up in the cCHIPS study. 160 HCMV-seropositive pregnant women were recruited. The definition of completing a time point (*T*) was the collection of at least 1 sample of saliva, urine, or vaginal secretions. The definition of completing the study was completing T4. Of the 8 withdrawn after T1, 7 were lost to follow-up (1 prior to and 6 during the COVID-19 restrictions) and 1 completed T1 out of window during the COVID-19 restrictions. Of the 14 who did not complete T2, 1 missed the appointment, 4 had their T1 visit during the T2 window, and 9 declined to freeze their samples at home during the COVID-19 restrictions. Of the 9 withdrawn after T2, 7 were lost to follow-up (4 prior to and 3 during the COVID-19 restrictions) and 2 completed T3 out of window during the COVID-19 restrictions. Of the 7 who did not complete T3, 6 declined to freeze their samples at home during the COVID-19 restrictions and 1 gave birth during the T3 window. Of the 28 withdrawn after T3, 26 were lost to follow-up (6 prior to and 20 during the COVID-19 restrictions) and 2 completed T4 out of window. Abbreviations: COVID-19, coronavirus disease 2019; HCMV, human cytomegalovirus.

Of the 3339 pregnant women approached for this study, 804 (24.1%) were tested for their HCMV serostatus (Figure 1). Of the 436 seropositive women (seroprevalence 54.2%), 160 (36.7%) were recruited into the study. In total, 83.5% (1604/1920) of saliva, urine and vaginal secretion samples were available for PCR testing, and 1591 results were available for analysis (Supplementary Figure 5).

### Prevalence, Quantity, and Natural History of HCMV Shedding

The prevalence of HCMV shedding in any bodily fluids in HCMV-seropositive women was 18.8% [30/160, 95% CI: 13.5–25.5%] during pregnancy (T1-T3), and 21.3% [34/160, 95% CI: 15.6–28.2%] during both pregnancy and the postpartum period (T1-T4) (Table 1). The prevalence of shedding either once, at consecutive time points, or intermittently in one or more bodily fluid during pregnancy and/or postpartum was 13.8% [22/160, 95% CI: 9.3–19.9%], 6.3% [10/160, 95% CI: 3.4–11.1%], and 1.3% [2/160, 95% CI: 0.3–4.4%], respectively (Supplementary Figure 6). Twenty-three women shed in 1 bodily fluid only [14.4%, 95% CI: 9.8–20.7%], and 11 women shed in more than 1 bodily fluid [6.9%, 95% CI: 3.9–11.9%] (Supplementary Figure 6).

**Table 1.** Prevalence and Quantity of HCMV Shedding According to Sample Type and Time Point for All Participants

Site		T1	T2	T3	Any T1–3	T4	Any T1–4
Vaginal secretions	# shedding /n	9/156	10/142	10/135	22/160	5/83	24/160
	% shedding	5.8%	7.0%	7.4%	13.8%	6.0**%**	15.0%
	[95% CI]	[3.1%, 10.6%]	[3.9%, 12.5%]	[4.1%, 13.1%]	[9.3%, 19.9%]	[2.6%, 13.3%]	[10.3%, 21.4%]
	Quantity (IU/ml)	800	189.5	640.5	…	260	…
	Median (LQ, UQ)	[199, 5450]	[96, 1407.8]	[202.8, 1550]	…	[188, 460.5]	…
Urine	# shedding /n	9/155	10/141	9/136	17/160	7/111	20/160
	% shedding	5.8%	7.1%	6.6%	10.6%	6.3%	12.5%
	[95% CI]	[3.1%, 10.7%]	[3.9%, 12.6%]	[3.5%, 12.1%]	[6.7%, 16.4%]	[3.1%, 12.5%]	[8.2%, 18.5%]
	Quantity (IU/ml)	31	119.5	31	…	31	…
	Median [LQ, UQ]	[31, 67]	[31, 733.25]	[31, 131.5]	…	[31, 389.5]	…
Saliva	# shedding /n	3/155	3/141	2/134	5/160	0/102	5/160
	% shedding	1.9%	2.1%	1.5%	3.1%	0.0%	3.1%
	[95% CI]	[0. 7%, 5.5%]	[0.7%, 6.1%]	[0.4%, 5.3%]	[1.4%, 7.1%]	[0.0%, 4.5%]	[1.4%, 7.1%]
	Quantity (IU/ml)	521	693	586.5	…	…	…
	Median [LQ, UQ]	[227, 689]	[402, 768]	[539, 634]	…	…	…
Any	# shedding /n	14/154	17/140	15/133	30/160	7/72	34/160
	% shedding	9.1%	12.1%	11.3%	18.8%	9.7%	21.3%
	[95% CI]	[5.5%, 14.7%]	[7.7%, 18.6%]	[7.0%, 17.8%]	[13.5%, 25.5%]	[4.8%, 18.7%]	[15.6%, 28.2%]

Abbreviations: #, frequency; CI, confidence intervals; HCMV, human cytomegalovirus; LQ, lower quartile; MSU, midstream urine; n, number of participants; T, time point; UQ, upper quartile.

At all-time points, the odds of shedding in urine were nearly the same as the odds of shedding in vaginal secretions [OR 1.02, 95% CI: 0.53–1.93, *P* = .961] (Table 2). The odds of shedding in saliva was significantly lower than the odds of shedding in vaginal secretions [OR 0.09, 95% CI: 0.03–0.26, *P* < .001] (Table 2), and remained significantly lower after adjustment for time point [AOR 0.09, 95% CI: 0.03–0.26, *P* < .001] (Supplementary Figure 7).

**Table 2.** Associations of Sample Type, Time Point, and Maternal Risk Factors With Detection of HCMV Shedding, Univariate, and Multivariable Mixed-effects Logistic Regression

		Univariate			Multivariable		
		OR	95% CI	*P* value	AOR	95% CI	*P* value
Sample							
Type	Vaginal secretions	Ref		…	Ref	…	…
	Urine	1.0	0.53, 1.93	.961	1.0	0.53, 2.00	.940
	Saliva	0.1	0.03, 0.26	<.001	0.1	0.04, 0.30	<.001
Time point	1	Ref		…	Ref	…	…
	2	1.2	0.58, 2.57	.606	1.2	0.54, 2.84	.610
	3	1.2	0.53, 2.48	.721	1.2	0.49, 2.70	.748
	4	1.0	0.40, 2.48	1.000	1.0	0.37, 2.78	.978
Demographics							
Age (y)	<30	Ref		…	…	…	…
	30–39	1.3	0.17, 9.94	.812	…	…	…
	>39	3.1	0.13, 70.6	.487	…	…	…
Ethnicity	White British	Ref		…	Ref	…	…
	White Other	0.2	0.05, 0.95	.043	0.3	0.03, 1.86	.177
	South Asian	0.3	0.05, 1.89	.208	0.3	0.03, 3.11	.298
	Asian Other	0.8	0.08, 8.64	.868	0.7	0.04, 9.92	.765
	Black	0.4	0.03, 4.94	.442	0.4	0.02, 12.20	.617
	Mixed/Other	0.0	0.00, *inf*	.903	0.0	0.0, *inf*	1.000
Born in UK	No	Ref		…	…	…	…
	Yes	1.3	0.36, 4.73	.693	…	…	…
Duration in UK (y)	<5	Ref		…	…	…	…
	5–15	1.4	0.12, 17.20	.785	…	…	…
	>15	1.4	0.13, 14.0	.792	…	…	…
Highest education	A-level/GCSE	Ref		…	…	…	…
	Diploma	4.0	0.41, 38.40	.235	…	…	…
	First degree	2.0	0.26, 15.40	.510	…	…	…
	PhD/Masters	1.4	0.17, 11.40	.756	…	…	…
No. of pregnancies	2	Ref		…	Ref	…	…
	3	2.2	0.55, 8.76	.267	1.8	0.37, 8.48	.471
	>3	0.2	0.03, 1.18	.073	0.2	0.01, 3.28	.243
No. of children	1	Ref		…	…	…	…
	2	1.2	0.20, 6.80	.873	…	…	…
	>2	1.2	0.07, 19.17	.908	…	…	…
Children aged 1 y	No	Ref		…	…	…	…
	Yes	0.7	0.19, 2.92	.677	…	…	…
Children aged 2 y	No	Ref		…	…	…	…
	Yes	2.3	0.64, 8.14	.204	…	…	…
Children aged 3 y	No	Ref		…	…	…	…
	Yes	0.4	0.07, 2.25	.288	…	…	…
Work regularly with children	No	Ref		…	…	…	…
	Yes	2.4	0.47, 12.20	.293	…	…	…
No. of households	≤3	Ref		…	…	…	…
	>3	0.9	0.20, 3.70	.839	…	…	…
Hygiene factors							
Washing hands with gel		1.1	0.63, 1.86	.766	…	…	…
Saliva contact		0.9	0.45, 1.71	.700	…	…	…
Washing hands with water only		0.8	0.44, 1.34	.353	…	…	…
Washing hands with soap		0.7	0.30, 1.75	.480	…	…	…

Only risk factors with a univariate association at a significance level of <0.1 (ethnicity and no. of pregnancies) were included in the multivariable model.

Abbreviations: A-level, advanced level; AOR, adjusted odds ratio; CI, confidence interval; GCSE, general certificate of secondary education; HCMV, human cytomegalovirus; inf, infinity; OR, odds ratio; PhD, Doctor of Philosophy; Ref, reference; UK, United Kingdom.

The quantity of shedding in urine was significantly lower [IRR 0.1, 95% CI: 0.05–0.28, *P* < .001] than in vaginal secretions (Table 3). The quantity of shedding in saliva was also lower than in vaginal secretions, though not statistically significant, despite a substantially higher and statistically significant odds of no shedding in saliva [OR 5.3, 95% CI: 2.31–12.10, *P* < .001] (Table 3). The associations remained unchanged after adjustment for time point (Table 3).

**Table 3.** Associations of Sample Type, Time point, and Maternal Risk Factors With Quantity of HCMV Shedding; Univariate and Multivariable Mixed-effects Zero-inflated Negative Binomial Regression

		Univariate						Multivariable					
		Zero Inflated Component			Conditional Component			Zero Inflated Component			Conditional Component		
		OR	95% CI	*P*	IRR	95% CI	*P*	AOR	95% CI	*P*	AIRR	95% CI	*P*
Sample													
Type	Vaginal secretions	Ref		…	Ref	…	…	Ref	…	…	Ref	…	…
	Urine	0.9	0.48, 1.50	.567	0.1	0.05, 0.28	<.001	0.8	0.47, 1.49	.546	0.1	0.06, 0.30	<.001
	Saliva	5.3	2.31, 12.10	<.001	0.4	0.09, 1.66	.197	5.4	2.34, 12.20	<.001	0.5	0.10, 1.88	.272
Time point	1	Ref		…	Ref	…	…	Ref	…	…	Ref	…	…
	2	0.8	0.42, 1.62	.574	0.9	0.28, 2.70	.812	0.9	0.42, 1.71	.649	1.6	0.60, 6.18	.357
	3	0.9	0.43, 1.74	.681	0.5	0.15, 1.47	.197	0.9	0.43, 1.81	.724	0.7	0.24, 1.76	.399
	4	1.1	0.50, 2.51	.786	0.1	0.04, 0.57	.006	1.3	0.53, 2.89	.577	0.5	0.15, 1.70	.273
Demographics													
Age (y)	<30	Ref		…	Ref	…	…	…	…	…	…	…	…
	30–39	1.2	0.49, 3.07	.662	1.2	0.02, 68.91	.919	…	…	…	…	…	…
	>39	3.0	0.81, 10.89	.100	6.4	0.02, 2012.42	.529	…	…	…	…	…	…
Ethnicity	White British	Ref		…	Ref	…	…	…	…	…	…	…	…
	White Other	3.7	1.55, 8.85	.003	1.0	0.03, 29.51	1.000	…	…	…	…	…	…
	South Asian	1.2	0.50, 2.89	.686	0.4	0.01, 28.55	.683	…	…	…	…	…	…
	Asian Other	2.6	0.85, 8.20	.093	2.4	0.02, 329.87	.721	…	…	…	…	…	…
	Black	4.6	0.98, 21.28	.053	2.2	0.01, 630.73	.781	…	…	…	…	…	…
	Mixed/Other	0	0.00, *inf*	.997	0.0	0.00, 183.35	.448	…	…	…	…	…	…
Born in UK	No	Ref		…	Ref	…	…	…	…	…	…	…	…
	Yes	0.5	0.32, 0.94	.030	0.7	0.06, 8.09	.749	…	…	…	…	…	…
Duration in UK (y)	<5	Ref		…	Ref	…	…	…	…	…	…	…	…
	5–15	0.6	0.18, 1.94	.384	0.9	0.01, 118.53	.969	…	…	…	…	…	…
	>15	0.4	0.14, 1.38	.160	0.6	0.01, 61.10	.840	…	…	…	…	…	…
Highest education	A-level/GCSE	Ref		…	Ref	…	…	…	…	…	…	…	…
	Diploma	0.2	0.06, 0.60	.005	0.6	0.01, 57.32	.818	…	…	…	…	…	…
	First degree	0.3	0.08, 0.83	.022	0.4	0.01, 31.92	.709	…	…	…	…	…	…
	PhD/Masters	0.5	0.14, 1.52	.205	0.6	0.01, 50.99	.837	…	…	…	…	…	…
No. of pregnancies	2	Ref		…	Ref	…	…	…	…	…	…	…	…
	3	0.5	0.30, 0.91	.021	0.9	0.06, 15.30	.967	…	…	…	…	…	…
	>3	1.2	0.31, 4.37	.829	0.2	0.00, 26.15	.513	…	…	…	…	…	…
No. of children	1	Ref		…	Ref	…	…	…	…	…	…	…	…
	2	0.4	0.21, 0.81	.010	0.6	0.02, 18.11	.784	…	…	…	…	…	…
	>2	1.2	0.33, 4.54	.773	1.4	0.01, 318.57	.907	…	…	…	…	…	…
Children aged 1 y	No	Ref		…	Ref	…	…	…	…	…	…	…	…
	Yes	1.1	0.60,1.84	.853	1.0	0.07, 13.48	.985	…	…	…	…	…	…
Children aged 2 y	No	Ref		…	Ref	…	…	…	…	…	…	…	…
	Yes	1.3	0.80, 2.26	.271	2.5	0.21, 30.77	.468	…	…	…	…	…	…
Children aged 3 y	No	Ref		…	Ref	…	…	…	…	…	…	…	…
	Yes	0.4	0.20, 0.94	.035	0.2	0.00, 9.37	.383	…	…	…	…	…	…
Work regularly with children	No	Ref		…	Ref	…	…	…	…	…	…	…	…
	Yes	0.4	0.25, 0.80	.007	0.9	0.04, 19.53	.928	…	…	…	…	…	…
No. of household	≤3	Ref		…	Ref	…	…	…	…	…	…	…	…
	>3	1.6	0.83, 2.94	.169	1.5	0.09, 24.58	.789	…	…	…	…	…	…
Hygiene factors													
Washing hands with gel		1.1	0.83, 1.56	.416	0.9	0.27, 2.91	.850	…	…	…	…	…	…
Saliva contact		0.7	0.73, 0.74	.000	2.0	1.69, 2.33	.000	…	…	…	…	…	…
Washing hands with water only		1.0	0.71, 1.41	.995	0.4	0.12, 1.06	.064	…	…	…	…	…	…
Washing hands with soap		0.7	NA	NA	2.1	NA	NA	…	…	…	…	…	…

Abbreviations: AIRR, adjusted incidence rate ratio; A-level, advanced level; AOR, adjusted odds ratio; CI, confidence interval; GCSE, general certificate of secondary education; HCMV, human cytomegalovirus; inf, infinity; IRR, incidence rate ratio; NA, not applicable; OR, odds ratio; *P*, *P* value; PhD, Doctor of Philosophy; Ref, reference; UK, United Kingdom.

### Risk Factors for HCMV Shedding

A statistically significant difference in behavior over time as derived from the hygiene-related contact questionnaire using principal component analysis was not observed (Table 4).

**Table 4.** Summary Statistics of Hygiene Factors by Time Point; Mean (SD), Minimum—Maximum Values

Factor	T1 (n = 154)	T2 (n = 121)	T3 (n = 113)	T4 (n = 74)	*P* Value
Washing hands with gel	2.0 (0.97) 1.0–5.0	2.0 (0.92) 1.0–4.7	1.9 (0.94) 1.0–5.0	2.0 (0.88) 1.0–5.0	.899
Saliva contact	2.8 (0.84) 1.0–4.2	2.7 (0.87) 1.0–4.2	2.7 (0.89) 1.0–4.8	2.5 (0.82) 1.0–4.6	.119
Washing hands with water only	2.1 (0.88) 1.0–5.0	2.2 (0.88) 1.0–5.0	2.2 (0.87) 1.0–5.0	2.2 (0.85) 1.0–5.0	.912
Washing hands with soap and water	2.7 (0.54) 1.0–3.7	2.6 (0.54) 1.0–3.7	2.7 (0.55) 1.0–3.7	2.7 (0.50) 1.3–3.7	.708

A higher mean score indicated a greater frequency of such behavior.

Abbreviations: SD, standard deviation; T, time point.

Ethnicity was identified as a risk factor associated with detection of HCMV shedding across the time points using univariate mixed-effects model [White Other; OR 0.2, 95% CI: 0.05–0.95, *P* = .043] (Table 2). Number of pregnancies was identified as a risk factor associated with detection of shedding in any bodily fluids at any time point using simple logistic regression [>3; OR 0.2, 95% CI: 0.05–0.94, *P* = .042] (Table 5). The odds of shedding in saliva remained significantly lower compared with vaginal secretions after adjustment for time point, ethnicity, and number of pregnancies [AOR 0.11, 95% CI: 0.04–0.30, *P* < .001] (Table 2). Contact with children's saliva was associated with quantity of shedding in bodily fluids [IRR 1.98, 95% CI: 1.69–2.33, *P* < .001] (Table 3).

**Table 5.** Associations of Risk Factors With the Presence of HCMV Shedding in any Sample Over Time point 1 to 4, Univariate Simple Logistic Regression

		n (%)	OR	95% CI	*P* Value
Demographics					
Age (y)	<30	20 (13%)	Ref		…
	30–39	130 (81%)	1.3	0.37, 5.0	.652
	>39	10 (6%)	3.8	0.65, 22.0	.139
Ethnicity	White British	69 (43%)	Ref		…
	White Other	39 (24%)	0.5	0.17, 1.32	.156
	South Asian	23 (14%)	0.3	0.05, 1.17	.079
	Asian Other	9 (6%)	1.3	0.30, 5.80	.717
	Black	8 (5%)	0.9	0.16, 4.73	.879
	Mixed/Other	12 (8%)	0	0	.999
Born in UK	No	70 (44%)	Ref		…
	Yes	90 (56%)	1.0	0.46, 2.18	1.000
Duration in UK (y)	<5	14 (9%)	Ref		…
	5–15	44 (28%)	1.8	0.34, 9.23	.501
	>15	102 (64%)	1.5	0.30, 7.07	.636
Highest education	A-level/GCSE	23 (14%)	Ref		…
	Diploma	28 (18%)	2.2	0.50, 9.81	.292
	First degree	59 (37%)	1.7	0.43, 6.69	.446
	PhD/Masters	50 (31%)	1.7	0.41, 6.74	.474
No. of pregnancies	2	88 (55%)	Ref		…
	3	35 (22%)	1.7	0.69, 4.00	.254
	>3	37 (23%)	0.2	0.05, 0.94	.042
No. of children	1	125 (78%)	Ref		…
	2	26 (16%)	1.0	0.33, 2.77	.929
	>2	9 (6%)	1.1	0.22, 5.84	.873
Children aged 1 y	No	104 (65%)	Ref		…
	Yes	56 (35%)	1.0	0.43, 2.18	.934
Children aged 2 y	No	88 (55%)	Ref		…
	Yes	72 (45%)	1.8	0.81, 3.85	.156
Children aged 3 y	No	128 (80%)	Ref		…
	Yes	32 (20%)	0.4	0.10, 1.24	.105
Work regularly with children	No	133 (83%)	Ref		…
	Yes	27 (17%)	1.5	0.58, 3.97	.401
No. of households	≤3	116 (73%)	Ref		…
	>3	44 (28%)	1.0	0.44, 2.46	.929
Hygiene factors					
Washing hands with gel		154	1.3	0.90, 1.98	.145
Saliva contact		154	0.7	0.46, 1.19	.210
Washing hands with water only		154	1.1	0.69, 1.68	.740
Washing hands with soap and water		154	1.8	0.81, 3.84	.149

Abbreviations: A-level, advanced level; CI, confidence interval; GCSE, general certificate of secondary education; HCMV, human cytomegalovirus; OR, odds ratio; *P*, *P* value; PhD, Doctor of Philosophy; Ref, reference; UK, United Kingdom.

### T-cell Immune Responses

Over the 4 time points, 55.6% (89/160) and 41.9% (67/160) of participants provided QuantiFERON-CMV and T-SPOT.CMV samples, respectively. Of the 89 participants who provided QuantiFERON-CMV samples, 28 provided 1 sample and 61 provided more than 1 sample (Supplementary Figure 8). For T-SPOT.CMV ELISPOT, 36 and 31 participants provided 1 and more than 1 sample, respectively (Supplementary Figure 9).

The prevalence of T-cell immune responses as assessed by QuantiFERON-CMV was between 55.6% and 75.0% (Table 6), and 25% to 33.3% of participants did not have a detectable IFNγ response. Conversely, the prevalence of T-cell immune responses as assessed using the T-SPOT.CMV assay was ∼100%. At each time point, the median quantity of IFNγ released in QuantiFERON-CMV was similar (range 2.1 to 3.0 IU/ml), and the median quantity of IFNγ released in T-SPOT.CMV was greater in culture media stimulated with pp65 antigen than with IE1 antigen.

**Table 6.** Prevalence and Quantity of HCMV-specific T-cell Immune Responses According to Assay Type and Time point

Assay		T1	T2	T3	T4
QFN*^a^*	# Positive/n	50/72	39/52	31/43	10/18
	% Positive [95% CI]	69.4**%** [57.3%, 79.5%]	75.0% [60.8%, 85.5%]	72.1% [56.1%, 84.2%]	55.6% [31.4%, 77.6%]
	Quantity (IU/ml) Median [LQ, UQ]	2.96 [0.75, 7.99]	2.80 [0.68, 6.00]	2.71 [0.89, 6.92]	2.12 [1.17, 4.12]
	# Negative/n	22/72	13/52	11/43	6/18
	% Negative [95% CI]	30.6**%** [20.5%, 42.7%]	25.0**%** [14.5%, 39.2%]	25.6% [14.0%, 41.5%]	33.3% [14.4%, 58.9%]
	Quantity (IU/ml) Median [LQ, UQ]	0.03 [0.00, 0.09]	0.03 [0.00, 0.07]	0.06 [0.01, 0.11]	0.01 [0.00, 0.02]
T-SPOT					
Global	# Positive/n	49/49	24/25	25/26	8/8
	% Positive [95% CI]	100% [90.9%, 100%]	96.0% [77.7%, 99.8%]	96.2**%** [78.4%, 99.8%]	100% [59.8%, 100%]
IE1 Ag	# Positive/n	44/49	22/25	22/26	7/8
	% Positive [95% CI]	89.8**%** [77.0%, 96.2%]	88.0% [67.7%, 96.9%]	84.6**%** [64.3%, 95.0%]	87.5% [46.7%, 99.3%]
	Quantity (SC) Median [LQ, UQ]	44 [11, 167]	93 [28, 177]	54 [16, 98]	42 [36, 78]
pp65 Ag	# Positive/n	49/49	24/25	25/26	8/8
	% Positive [95% CI]	100% [90.9%, 100%]	96.0**%** [77.7%, 99.8%]	96.2**%** [78.4%, 99.8%]	100**%** [59.8%, 100%]
	Quantity (SC) Median [LQ, UQ]	149 [73, 260]	135 [100, 199]	146 [53, 243]	93 [49, 137]

Abbreviations: CI, confidence interval; HCMV, human cytomegalovirus; IE1 Ag, intermediate-early 1 antigen; LQ, lower quartile; n, number of participants; pp65 Ag, phosphoprotein 65 antigen; QFN, QuantiFERON-CMV ELISA; SC, spot count; T, time point; T-SPOT, T-SPOT.CMV ELISPOT; UQ, upper quartile.

^*a*^One participant had an indeterminate result due to a low mitogen response (<0.5 IU/ml) at T3 and T4. One participant had an indeterminate result due to a low mitogen response at T4.

Using QuantiFERON-CMV, T-cells immune responses for women who had more than 1 sample were mainly always positive or always negative (Supplementary Figure 8, qualitative results). For most women, the magnitude of quantitative IFNγ responses remained similar over time (Supplementary Figure 8, quantitative results). Neither qualitative (positive or negative) nor quantitative (low, medium, or high level) IFNγ responses appeared to consistently correlate with HCMV shedding or not shedding (Supplementary Figure 8, PCR data).

Using T-SPOT.CMV, T-cell immune responses were always positive for almost all women who had more than 1 sample (Supplementary Figure 9, qualitative results). Unlike QuantiFERON-CMV, a persistently negative T-cell immune response was not observed using the T-SPOT.CMV assay. Similar to QuantiFERON-CMV, the magnitude of IFNγ responses remained similar over time for most women, and neither qualitative nor quantitative IFNγ responses from either IE1 or pp65 appeared to consistently correlate with shedding or not shedding (Supplementary Figure 9). The median spot counts were always higher for pp65 than that for IE1.

While there was weak evidence that a lower pp65 count, as measured by T-SPOT.CMV, was associated with higher odds of shedding detection across all time points [OR 0.23, 95% CI: 0.05–1.23, *P* = .055], this was not statistically significant (Table 7). T-cell immune responses as measured by T-SPOT.CMV using IE1 and QuantiFERON-CMV were not associated with shedding detection.

**Table 7.** Association of HCMV-specific T-cell Immune Responses, as Quantitatively Measured by QuantiFERON-CMV and T-SPOT.CMV IGRAs, With HCMV Shedding Detection in any Sample Type, Mixed-effects Logistic Regression

IGRA	n, Observations	OR (95% CI)*^a^*	*P* Value
QuantiFERON-CMV	88, 179	1.01 (0.88, 1.16)	.918
T-SPOT.CMV: IE1	66, 104	1.00 (0.99, 1.01)	.866
T-SPOT.CMV: pp65	66, 104	0.23 (0.05, 1.03)	.055

QuantiFERON-CMV measures IFN-γ as international units/ml. T-SPOT.CMV measures IFN-y producing T-cells as spot-forming colonies produced per 250 000 PBMCs according to 2 HCMV-specific antigens, pp65 and IE1, separately. HCMV shedding was detected using quantitative PCR in saliva, urine, and/or vaginal secretion samples.

Abbreviations: CI, confidence interval; HCMV, human cytomegalovirus; IE1, intermediate-early 1 antigen; IGRA, interferon gamma release assay, n, number of samples, OR, odds ratio; *P*, *P* value; PBMC, peripheral blood mononuclear cell; pp65, phosphoprotein 65 antigen.

^*a*^Variable standardized to improve model identifiability.

The agreement between the QuantiFERON-CMV and T-SPOT.CMV for detecting T-cell immune responses during pregnancy and postpartum was 72.0% [77/107, 95% CI: 62.8–79.6] (Supplementary Figure 10). The agreement remained similar when results were stratified by shedding status (Supplementary Figure 1^1^).

## DISCUSSION

The prevalence of HCMV shedding in seropositive women during pregnancy and postpartum in this study was ∼20%, similar to the results of our recent meta-analysis of HCMV shedding during pregnancy (22%) [29]. The maternal seroprevalence observed here (54%) was also consistent with a recently completed study in London [30], and with a meta-analysis of seroprevalence in women of reproductive age in the United Kingdom [31].

Approximately 80% of HCMV-seropositive pregnant women in our study did not shed HCMV, marginally more than what was found in studies performed in Brazil (60%; seroprevalence 98%) [27] and Italy (65%; seroprevalence 68%) [32]. For those that shed, approximately two-thirds of participants shed only once, and in only 1 bodily fluid. Like the Italian study [32], our study found shedding prevalence to be highest in vaginal secretions and lowest in saliva, while the quantity of shedding did not significantly differ between these fluids. A change in shedding prevalence over time in any bodily fluids was not observed. It was noteworthy that the prevalence of shedding has also been reported to be higher in vaginal secretions than saliva or urine in postpartum and nonpregnant women in other studies [33, 34]. In agreement with the Brazilian study [27], shedding prevalence in our cohort did not vary during pregnancy, and was similar between vaginal secretions and urine. In contrast, the Brazilian group reported that shedding prevalence was highest in saliva. However, it is challenging to directly compare shedding prevalence between studies for several reasons. First, because of the different populations and demographics [27, 32–36]. Second, the hygiene practices and frequency of contact with children in these studies may be different. Third, HCMV IgG avidity and IgM tests were not performed in some studies [27, 35] and therefore, the inclusion of seropositive women with a recent primary infection cannot be excluded [12]. Finally, variations in, and a lack of full reporting of, sampling and laboratory methods make comparison of studies problematic [27, 32–34, 36]. Our recent studies of HCMV DNA detection have demonstrated that the detection and recovery of HCMV DNA are dependent on fluid type, the use of validated collection devices and preservation media, plus the duration and temperature of storage [22–24]. As DNA degradation postcollection does occur [22, 23, 37], it is imperative to utilize validated sampling, preanalytical, and analytical methods in shedding studies. Therefore, it is possible that any differences in detection of HCMV DNA between studies were the direct result of how the samples were handled or that HCMV DNA has degraded before detection.

Consistent with the Italian cohort, we found that maternal age and close contact with children or children's bodily fluids were not risk factors for shedding [32], although we found that contact with children's saliva, specifically, was associated with a higher quantity of shedding. This may reflect a higher HCMV DNA level in saliva compared with other fluids [38, 39]. A Brazilian study found that exposure to children aged 3–6 years and household crowding were associated with shedding [27]. These different associations may reflect the different populations studied. That said, our observations indicate that ethnicity and gravidity are likely to be important risk factors in HCMV shedding. Inaccurate reporting of, and a change in, hygiene practice or behavior during the pandemic may also have confounded our results [40–42].

The agreement between the QuantiFERON-CMV and T-SPOT.CMV for detecting T-cell immune responses during pregnancy and postpartum in HCMV-seropositive women was ∼70%. To our knowledge, no other study has made this comparison. The prevalence and quantity of T-cell immune responses over time varied between assays. Overall, IFNγ responses did not appear to correlate with shedding, although there is relatively weak evidence of an association between a lower pp65 count and higher odds of HCMV shedding. Studies with larger sample sizes should be undertaken to investigate this further.

Additionally, further caution should be exercised when comparing IGRAs between different studies. Results of IGRAs are not interchangeable and should not be compared due to a lack of assay standardization and methodological differences [14–16, 21, 43–45]. Importantly, a lack of IFNγ responses in IGRAs in HCMV-seropositive women is not uncommon and may be due to the use of synthetic peptides or uncommon HLA types not detected by the assays, plus the inherent heterogeneity of different individuals immune responses to HCMV [43, 45, 46]. These factors may contribute to the disagreement between QuantiFERON-CMV and T-SPOT.CMV results we found in our current study.

The prevalence and natural history of HCMV shedding in HCMV-seropositive women determined in this study may contribute to the development of future disease burden models and therapeutic or preventative strategies against cCMV infection in the context of nonprimary maternal infection. The inconsistent association between maternal risk factors and shedding found in our study and previous reports requires further evaluation and would benefit from larger studies and the addition of HCMV genomic sequencing to accurately determine the molecular basis of HCMV interaction with the host. Moreover, further work is required to investigate the utility of IGRAs for studying T-cell immune responses in HCMV-seropositive women. The findings from our current study highlight the need for future larger-scale trials from diverse populations to investigate the relationship between HCMV shedding, T-cell immune responses and their association with cCMV in HCMV-seropositive pregnant women.

## Supplementary Material

ciag076_Supplementary_Data

## Supplementary Data

Supplementary materials are available at *Clinical Infectious Diseases* online. Consisting of data provided by the authors to benefit the reader, the posted materials are not copyedited and are the sole responsibility of the authors, so questions or comments should be addressed to the corresponding author.

## References

[1] Korndewal MJ, Oudesluys-Murphy AM, Kroes ACM, van der Sande MAB, de Melker HE, Vossen ACTM. Long-term impairment attributable to congenital Cytomegalovirus infection: a retrospective cohort study. Dev Med Child Neurol 2017; 59:1261–8. 28990181 10.1111/dmcn.13556

[2] Retzler J, Hex N, Bartlett C, et al Economic cost of congenital CMV in the UK. Arch Dis Child 2019; 104:559–63. 30472664 10.1136/archdischild-2018-316010

[3] Luck SE, Wieringa JW, Blazquez-Gamero D, et al Congenital Cytomegalovirus: a European expert consensus statement on diagnosis and management. Pediatr Infect Dis J 2017; 36:1205–13. 29140947 10.1097/INF.0000000000001763

[4] Boppana SB, Rivera LB, Fowler KB, Mach M, Britt WJ. Intrauterine transmission of cytomegalovirus to infants of women with preconceptional immunity. N Engl J Med 2001; 344:1366–71. 11333993 10.1056/NEJM200105033441804

[5] Fowler KB, Stagno S, Pass RF, Britt WJ, Boll TJ, Alford CA. The outcome of congenital Cytomegalovirus infection in relation to maternal antibody status. N Engl J Med 1992; 326:663–7. 1310525 10.1056/NEJM199203053261003

[6] Yamamoto AY, Mussi-Pinhata MM, Boppana SB, et al Human Cytomegalovirus reinfection is associated with intrauterine transmission in a highly Cytomegalovirus-immune maternal population. Am J Obstet Gynecol 2010; 202:297.e1–e8. 10.1016/j.ajog.2009.11.018 PMC8351475 20060091

[7] Cannon MJ, Hyde TB, Schmid DS. Review of Cytomegalovirus shedding in bodily fluids and relevance to congenital Cytomegalovirus infection. Rev Med Virol 2011; 21:240–55. 21674676 10.1002/rmv.695 PMC4494736

[8] Kenneson A, Cannon MJ. Review and meta-analysis of the epidemiology of congenital cytomegalovirus (CMV) infection. Rev Med Virol 2007; 17:253–76. 17579921 10.1002/rmv.535

[9] Chatzakis C, Ville Y, Makrydimas G, Dinas K, Zavlanos A, Sotiriadis A. Timing of primary maternal Cytomegalovirus infection and rates of vertical transmission and fetal consequences. Am J Obstet Gynecol 2020; 223:870–83.e11. 32460972 10.1016/j.ajog.2020.05.038

[10] Faure-Bardon V, Millischer AE, Deloison B, et al Refining the prognosis of fetuses infected with Cytomegalovirus in the first trimester of pregnancy by serial prenatal assessment: a single-centre retrospective study. BJOG 2020; 127:355–62. 31505103 10.1111/1471-0528.15935

[11] de Vries JJ, van Zwet EW, Dekker FW, Kroes ACM, Verkerk PH, Vossen ACTM. The apparent paradox of maternal Seropositivity as a risk factor for congenital Cytomegalovirus infection: a population-based prediction model. Rev Med Virol 2013; 23:241–9. 23559569 10.1002/rmv.1744

[12] Leruez-Ville M, Chatzakis C, Lilleri D, et al Consensus recommendation for prenatal, neonatal and postnatal management of congenital cytomegalovirus infection from the European Congenital Infection Initiative (ECCI). Lancet Reg Health Eur 2024; 40:100892. 38590940 10.1016/j.lanepe.2024.100892 PMC10999471

[13] Mussi-Pinhata MM, Yamamoto AY. Natural history of congenital Cytomegalovirus infection in highly seropositive populations. J Infect Dis 2020; 221:S15–22. 32134482 10.1093/infdis/jiz443 PMC7057789

[14] Saldan A, Forner G, Mengoli C, et al Comparison of the cytomegalovirus (CMV) enzyme-linked immunosorbent spot and CMV QuantiFERON cell-mediated immune assays in CMV-seropositive and -seronegative pregnant and nonpregnant women. J Clin Microbiol 2016; 54:1352–6. 26962091 10.1128/JCM.03128-15 PMC4844750

[15] Saldan A, Forner G, Mengoli C, Gussetti N, Palù G, Abate D. Strong cell-mediated immune response to human Cytomegalovirus is associated with increased risk of fetal infection in primarily infected pregnant women. Clin Infect Dis 2015; 61:1228–34. 26175520 10.1093/cid/civ561

[16] Eldar-Yedidia Y, Bar-Meir M, Hillel M, et al Low interferon relative-response to Cytomegalovirus is associated with low likelihood of intrauterine transmission of the virus. PLoS One 2016; 11:e0147883. 26881863 10.1371/journal.pone.0147883 PMC4755570

[17] Fornara C, Cassaniti I, Zavattoni M, et al Human Cytomegalovirus-specific memory CD4+ T-cell response and its correlation with virus transmission to the Fetus in pregnant women with primary infection. Clin Infect Dis 2017; 65:1659–65. 29020188 10.1093/cid/cix622

[18] Fornara C, Furione M, Arossa A, Gerna G, Lilleri D. Comparative magnitude and kinetics of human Cytomegalovirus-specific CD4(+) and CD8(+) T-cell responses in pregnant women with primary versus remote infection and in transmitting versus non-transmitting mothers: its utility for dating primary infection in pregnancy. J Med Virol 2016; 88:1238–46. 26680747 10.1002/jmv.24449

[19] Fornara C, Lilleri D, Revello MG, et al Kinetics of effector functions and phenotype of virus-specific and gammadelta T lymphocytes in primary human Cytomegalovirus infection during pregnancy. J Clin Immunol 2011; 31:1054–64. 21847524 10.1007/s10875-011-9577-8

[20] Soriano-Ramos M, Esquivel-De la Fuente E, Albert Vicent E, et al The role of the T-cell mediated immune response to Cytomegalovirus infection in intrauterine transmission. PLoS One 2023; 18:e0281341. 36745589 10.1371/journal.pone.0281341 PMC9901742

[21] Forner G, Saldan A, Mengoli C, Gussetti N, Palù G, Abate D. Cytomegalovirus (CMV) enzyme-linked immunosorbent spot assay but not CMV QuantiFERON assay is a novel biomarker to determine risk of congenital CMV infection in pregnant women. J Clin Microbiol 2016; 54:2149–54. 27280418 10.1128/JCM.00561-16 PMC4963489

[22] Tan NK, Pope CF, Carrington D. Screening for Cytomegalovirus shedding in vagina and saliva: significant differences between biological fluids, swab types and storage durations in DNA recovery. J Clin Virol 2022; 146:105055. 34953320 10.1016/j.jcv.2021.105055

[23] Tan NK, Carrington D, Pope CF. Detecting human cytomegalovirus in urine, vagina and saliva: impact of biological fluids and storage durations and temperatures on CMV DNA recovery. J Med Virol 2023; 95:e29081. 37675875 10.1002/jmv.29081

[24] Tan NK, Pope CF, Carrington D. Performance evaluation of fully automated cobas(R) 6800 CMV PCR for the detection and quantification of Cytomegalovirus DNA in neonatal urine and saliva, and adult urine, saliva, and vaginal secretion. J Med Virol 2023; 95:e29223. 37966419 10.1002/jmv.29223

[25] Qiagen . QuantiFERON-CMV ELISA package insert. 1075110 Rev. 05 ed. Hilden, Germany: Qiagen GmbH; **2018**:1–52.

[26] Oxford_Immunotec . T-SPOT.CMV package insert. PI-CMV-IVD-UK-V3 ed. Abingdon, UK: Oxford Immunotec Ltd; **2023**:1–12.

[27] Barbosa NG, Yamamoto AY, Duarte G, et al Cytomegalovirus shedding in seropositive pregnant women from a high-Seroprevalence population: the Brazilian cytomegalovirus hearing and maternal secondary infection study. Clin Infect Dis 2018; 67:743–50. 29490030 10.1093/cid/ciy166 PMC6094000

[28] Harris PA, Taylor R, Thielke R, Payne J, Gonzalez N, Conde JG. Research electronic data capture (REDCap)--a metadata-driven methodology and workflow process for providing translational research informatics support. J Biomed Inform 2009; 42:377–81. 18929686 10.1016/j.jbi.2008.08.010 PMC2700030

[29] Sapuan S, Theodosiou AA, Strang BL, Heath PT, Jones CE. A systematic review and meta-analysis of the prevalence of human Cytomegalovirus shedding in seropositive pregnant women. Rev Med Virol 2022; 32:e2399. 36196755 10.1002/rmv.2399 PMC9786761

[30] Calvert A, Vandrevala T, Parsons R, et al Changing knowledge, attitudes and behaviours towards cytomegalovirus in pregnancy through film-based antenatal education: a feasibility randomised controlled trial of a digital educational intervention. BMC Pregnancy Childbirth 2021; 21:565. 34407771 10.1186/s12884-021-03979-z PMC8375137

[31] Zuhair M, Smit GSA, Wallis G, et al Estimation of the worldwide Seroprevalence of Cytomegalovirus: a systematic review and meta-analysis. Rev Med Virol 2019; 29:e2034. 30706584 10.1002/rmv.2034

[32] Zelini P, d'Angelo P, De Cicco M, et al Human Cytomegalovirus non-primary infection during pregnancy: antibody response, risk factors and newborn outcome. Clin Microbiol Infect 2022; 28:1375–81. 34555536 10.1016/j.cmi.2021.09.013

[33] Azenkot T, Zaniello B, Green ML, et al Cytomegalovirus shedding from breastmilk and mucosal sites in healthy postpartum women: a pilot study. J Med Virol 2019; 91:894–8. 30578684 10.1002/jmv.25386 PMC6402967

[34] Ju D, Li XZ, Shi YF, Li Y, Guo LQ, Zhang Y. Cytomegalovirus shedding in seropositive healthy women of reproductive age in Tianjin, China. Epidemiol Infect 2020; 148:e34. 32070447 10.1017/S0950268820000217 PMC7058649

[35] Gatta LA, Rochat E, Weber JM, et al Clinical factors associated with Cytomegalovirus shedding among Seropositive pregnant women. Am J Obstet Gynecol MFM 2022; 4:100560. 34990874 10.1016/j.ajogmf.2021.100560 PMC9942897

[36] Huang Y, Guo X, Song Q, et al Cytomegalovirus shedding in healthy Seropositive female college students: a 6-month longitudinal study. J Infect Dis 2018; 217:1069–73. 29294037 10.1093/infdis/jix679

[37] Lilleri D, Tassis B, Pugni L, et al Prevalence, outcome, and prevention of congenital Cytomegalovirus infection in neonates born to women with preconception immunity (CHILd study). Clin Infect Dis 2023; 76:513–20. 35717635 10.1093/cid/ciac482 PMC9907511

[38] Stowell JD, Mask K, Amin M, et al Cross-sectional study of Cytomegalovirus shedding and immunological markers among Seropositive children and their mothers. BMC Infect Dis 2014; 14:568. 25388365 10.1186/s12879-014-0568-2 PMC4236433

[39] Cannon MJ, Stowell JD, Clark R, et al Repeated measures study of weekly and daily Cytomegalovirus shedding patterns in saliva and urine of healthy Cytomegalovirus-Seropositive children. BMC Infect Dis 2014; 14:569. 25391640 10.1186/s12879-014-0569-1 PMC4240830

[40] Villaverde S, Esquivel E, Baquero-Artigao F, et al Impact of the COVID-19 pandemic on the diagnosis of congenital Cytomegalovirus infection in Spain. Pediatr Infect Dis J 2022; 41:590–2. 35363648 10.1097/INF.0000000000003532 PMC9177127

[41] Schleiss MR, Rosendahl S, McCann M, Dollard SC, Lanzieri TM. Assessment of congenital Cytomegalovirus prevalence among newborns in Minnesota during the COVID-19 pandemic. JAMA Netw Open 2022; 5:e2230020. 36053537 10.1001/jamanetworkopen.2022.30020 PMC9440402

[42] Toriyabe K, Kitamura A, Hagimoto-Akasaka M, et al Transient decrease in incidence rate of maternal primary Cytomegalovirus infection during the COVID-19 pandemic in Japan. Viruses 2023; 15:1096. 37243182 10.3390/v15051096 PMC10222815

[43] Reuschel E, Barabas S, Zeman F, et al Functional impairment of CMV-reactive cellular immunity during pregnancy. J Med Virol 2017; 89:324–31. 27447923 10.1002/jmv.24639

[44] Callens R, Colman S, Delie A, et al Immunologic monitoring after allogeneic stem cell transplantation: T-SPOT.CMV and QuantiFERON-CMV, are they the same? Transplant Cell Ther 2023; 29:392.e1–e7. 10.1016/j.jtct.2023.03.018 36963722

[45] Abate D, Saldan A, Forner G, Tinto D, Bianchin A, Palù G. Optimization of interferon gamma ELISPOT assay to detect human Cytomegalovirus specific T-cell responses in solid organ transplants. J Virol Methods 2014; 196:157–62. 24216234 10.1016/j.jviromet.2013.10.036

[46] Valle-Arroyo J, Aguado R, Paez-Vega A, et al Lack of Cytomegalovirus (CMV)-specific cell-mediated immune response using QuantiFERON-CMV assay in CMV-Seropositive healthy volunteers: fact not artifact. Sci Rep 2020; 10:7194. 32346028 10.1038/s41598-020-64133-x PMC7188901

