# Supplementary material for: Human Cytomegalovirus (HCMV) Shedding and T-cell Immune Responses in HCMV-seropositive Women During Pregnancy and Postpartum: Prevalence, Natural History, and Risk Factors
Source: Clin Infect Dis. 2026 Feb 16;82(5):e1072–82. doi: 10.1093/cid/ciag076 (PMC13189655; doi:10.1093/cid/ciag076)
Supplement: ciag076_Supplementary_Data [file ciag076_supplementary_data.docx]

**Supplemental data**

*Supplementary Figure 1*

*
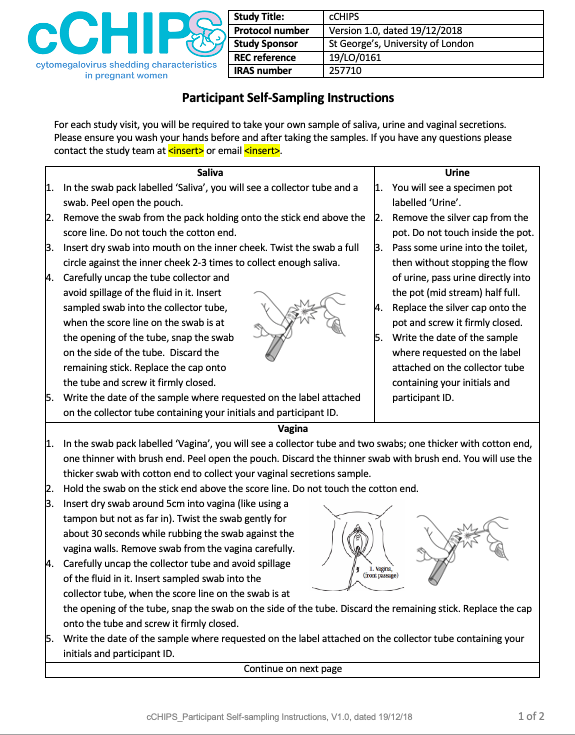
*

*
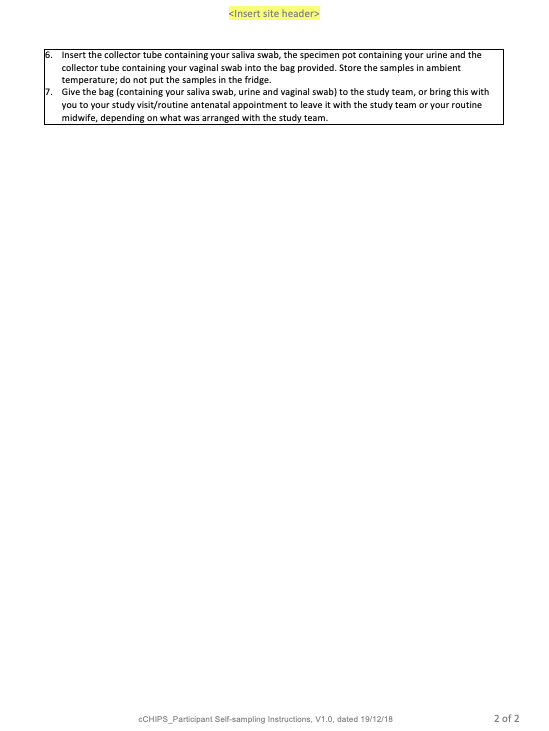
*

*Supplementary Figure 2*


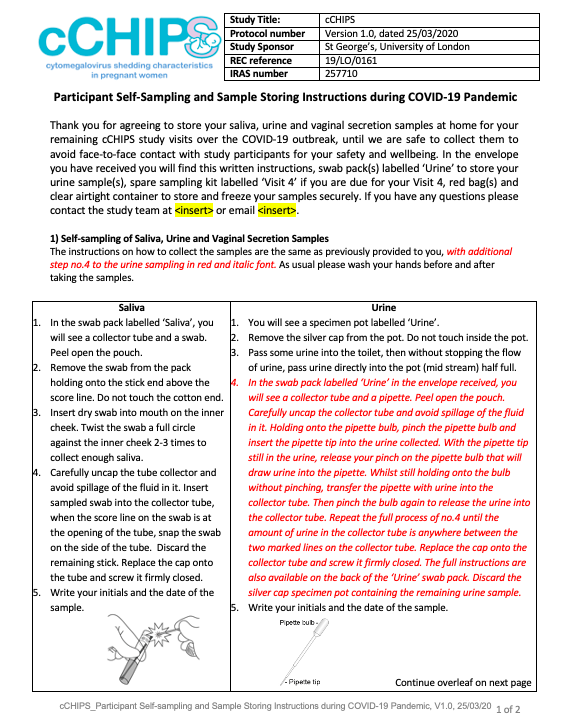


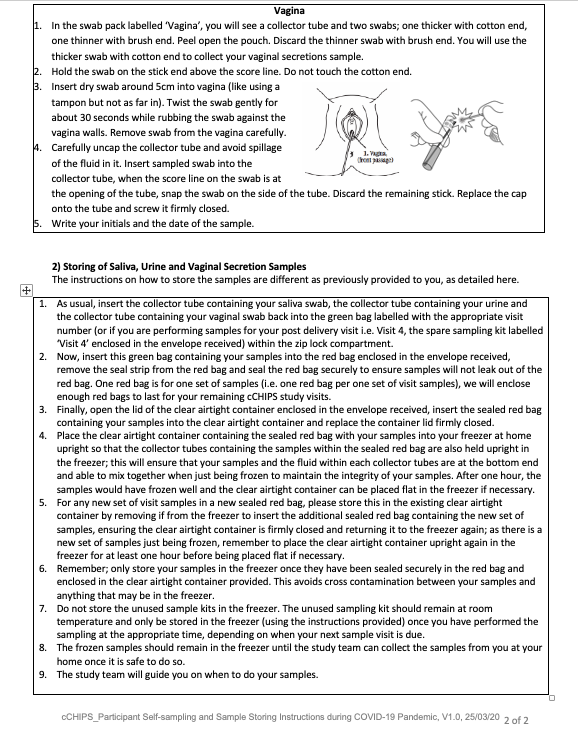


*Supplementary Figure 3*


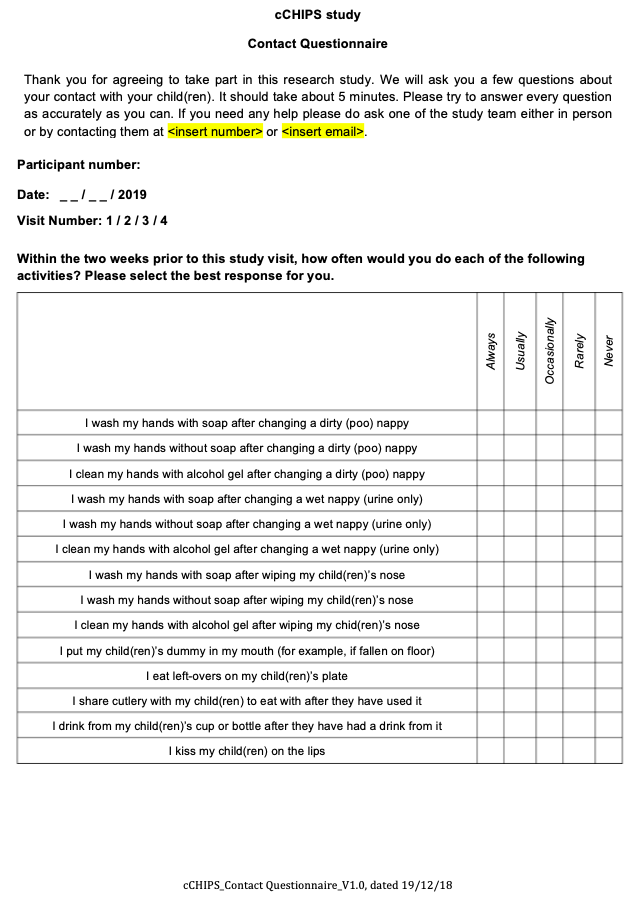


*Supplementary Figure 4*

Principal component analysis with Varimax rotation to derive four hygiene-related factors contact with children’s bodily

| Handling/contact with children’s bodily fluids | Washing hands with gel | Saliva contact | Washing hands with water only | Washing hands with soap and water |
| --- | --- | --- | --- | --- |
| gelfaeces1 | 0.927 | -0.016 | -0.042 | -0.040 |
| gelurine1 | 0.921 | -0.033 | -0.039 | 0.015 |
| gelnose1 | 0.856 | -0.014 | 0.090 | 0.204 |
| sharecutlery1 | -0.103 | 0.856 | 0.123 | 0.053 |
| eatleftover1 | -0.180 | 0.777 | 0.112 | 0.064 |
| sharecup1 | -0.006 | 0.763 | 0.206 | 0.063 |
| dummymouth1 | 0.187 | 0.491 | -0.219 | -0.180 |
| kisslips1 | 0.121 | 0.481 | -0.206 | -0.357 |
| nosoapurine1 | -0.041 | -0.067 | 0.728 | -0.268 |
| nosoapfaeces1 | 0.037 | 0.161 | 0.695 | -0.225 |
| nosoapnose1 | 0.028 | 0.062 | 0.693 | 0.161 |
| soapnose1 | 0.389 | 0.002 | 0.093 | 0.651 |
| soapfaeces1 | 0.193 | -0.077 | 0.270 | -0.643 |
| soapurine1 | 0.297 | -0.132 | -0.337 | 0.554 |

Principal component analysis was employed to analyse the frequency of handling and contact with child(ren)’s faeces, urine and saliva data obtained from 14 Likert style questions. Only data at timepoint 1 (T1) was entered into the principal component analysis to explore if the information it contains can be explained by fewer dimensions/factors. T1 was where the most data were available therefore most likely to identify a robust solution.

Four factors were extracted (all had a determinant > 1) and explained 62% of the total variation. A Varimax rotation was applied to the four factors and the factor loadings sorted according to size. It can be seen that the four factors are easily interpretable as ‘washing hands with gel’, ‘saliva contact’, ‘washing hands with water only’, and ‘washing hands with soap and water’. One item ‘washing hands with soap and water’ after handling child faeces was negatively loaded therefore the Likert scores were reversed prior to calculating the four domains as the mean value of items assigned to each domain. Using this domain structure the mean scores were also calculated for T2-T4.

*Supplementary Figure 5*

Number of HCMV shedding samples received and tested in the cCHIPS study.

Storage of samples at -20°C (instead of -80°C) during the COVID-19 pandemic did not appear to affect HCMV DNA stability and recovery. This is likely to be due to the use of DNA preservation media and a relatively short duration of pre-analytical handling and transport prior to long-term storage. See text for further details.


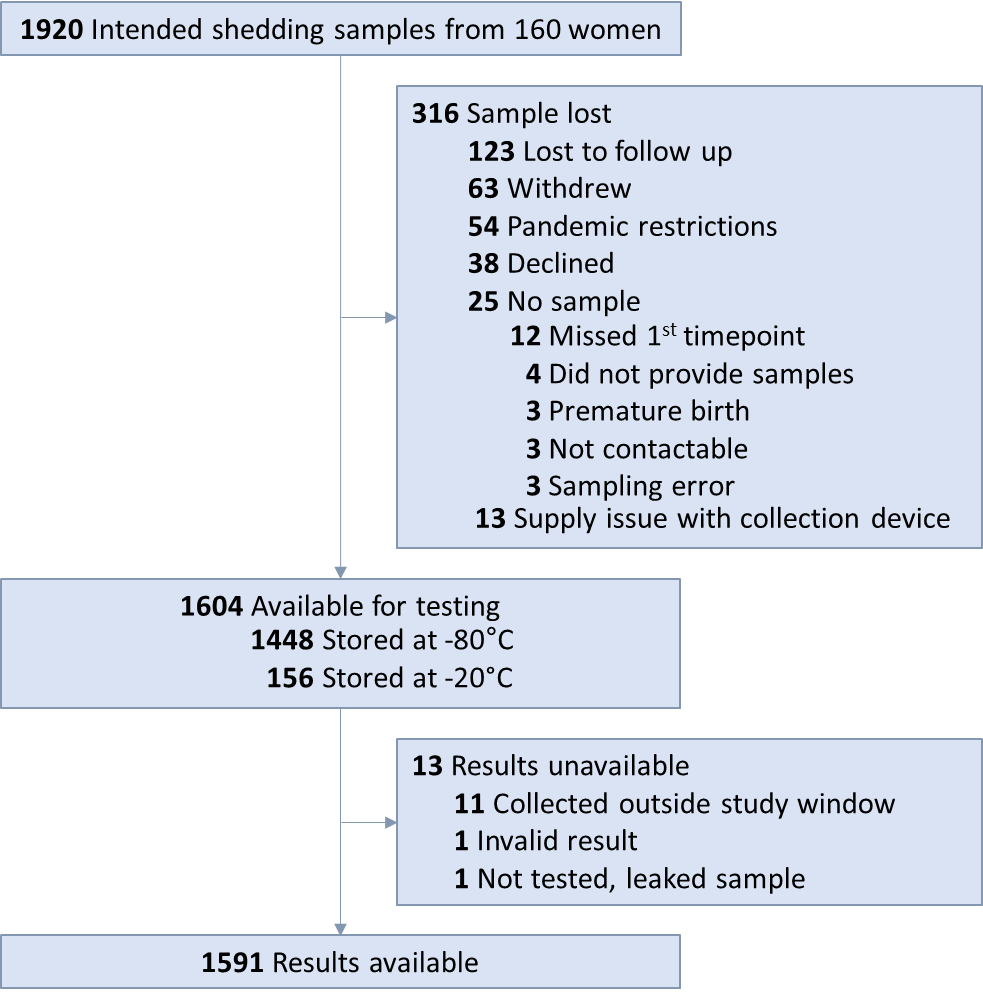


| Women | T1 | T2 | T3 | T4 |
| --- | --- | --- | --- | --- |
| 1 | ▲521, ●10900, ◆32000 | ▲402, ●4930, ◆3660 | ●373, ◆6480 | ●2490, ◆282 |
| 2 |  |  | ◆106 |  |
| 3 |  |  | ◆398 | ●31, ◆639 |
| 4 |  |  |  | ◆210 |
| 5 |  | ◆170 | ◆353 | -SUV |
| 6 | ◆439 |  | ●31 |  |
| 7 |  | ◆81 |  |  |
| 8 |  |  | ◆1010 | -V |
| 9 |  | ◆81 |  |  |
| 10 | ◆132 |  |  |  |
| 11 |  |  | ●31 | -V |
| 12 |  | ●31, ◆209 | ●31, ◆883 | -SUV |
| 13 | ●31 | ●208 | ●31 | ●31 |
| 14 |  |  |  | ●31 |
| 15 | ●31 |  |  | -SUV |
| 16 | ◆133 | ▲768, ●601 | ▲634, ●31 | -SUV |
| 17 |  | ●31 |  | -SUV |
| 18 | ●31, ◆9630 | ●1130 | ●232, ◆947 | -SUV |
| 19 |  |  | ◆163 |  |
| 20 |  | ◆103 |  | -SUV |
| 21 | ●31, ◆265 | ▲693 | ▲539, ●31 | -SUV |
| 22 |  |  | ◆216 |  |
| 23 | ▲227, ◆1270 | ●339, ◆7500 | ●31, ◆3170 | ●439, ◆166 |
| 24 | ▲689, ●31 |  | -SUV | -SUV |
| 25 |  | -U | -SUV | ●241, -S |
| 26 | ●135 |  |  |  |
| 27 |  | ●31 |  |  |
| 28 | ◆800 | -SUV | -SUV |  |
| 29 |  | ◆302 |  |  |
| 30 |  |  |  | ◆260, -S |
| 31 | ●31 | ●31 |  | -S |
| 32 |  | ●31, ◆657 |  | -V |
| 33 | ●399, ◆1270 |  |  | ●31 |
| 34 | -SUV | ◆101 |  | -V |

*Supplementary Figure 6*

Natural history of HCMV shedding and quantity (in IU/ml) in saliva (S, ▲), urine (U, ●), and vaginal secretions (V, ◆) of 34 HCMV-seropositive women with HCMV shedding detection in any bodily fluid at any timepoint (T1, 12-16 GW; T2, 17-26 GW; T3, ≥27 GW to pre-delivery; T4, ≤6 weeks post-delivery). Urine and vaginal secretion samples that had HCMV DNA detected but could not be quantified by PCR were assigned 31 and 81 IU/ml (limit of detection), respectively. Blank cells signify no shedding detection in all sample types. ‘-‘ denotes missing sample(s) e.g., -V, missing vaginal secretions sample.

Abbreviations: DNA, deoxyribonucleic acid; GW, gestational weeks; HCMV, human cytomegalovirus; IU/ml, international unit per millilitre; PCR, polymerase chain reaction; S, saliva; T, timepoint; U, urine; V, vaginal secretions.

*Supplementary Figure 7*

Associations of sample type and timepoint with presence of HCMV shedding, univariate and multivariable mixed-effects logistic regression

|  | | **Univariate** | | | **Multivariable** | | |
| --- | --- | --- | --- | --- | --- | --- | --- |
|  |  | **OR** | **95% CI** | **p-value** | **AOR** | **95% CI** | **p-value** |
| **Sample** | |  |  |  |  |  |  |
| Type | Vaginal secretions | ref |  |  | ref |  |  |
|  | Urine | 1.0 | 0.53, 1.93 | 0.961 | 1.0 | 0.54, 1.94 | 0.949 |
|  | Saliva | 0.1 | 0.03, 0.26 | <0.001 | 0.1 | 0.03, 0.26 | <0.001 |
| Timepoint | 1 | ref |  |  | ref |  |  |
|  | 2 | 1.2 | 0.58, 2.57 | 0.606 | 1.3 | 0.56, 2.77 | 0.590 |
|  | 3 | 1.2 | 0.53, 2.48 | 0.721 | 1.2 | 0.51, 2.65 | 0.718 |
|  | 4 | 1.0 | 0.40, 2.48 | 1.000 | 1.0 | 0.38, 2.61 | 0.990 |

*Supplementary Figure 8*

Natural history of T-cell immune responses during pregnancy (T1 to T3) and postpartum (T4) as assessed by QuantiFERON-CMV ELISA for 61 women who had more than one QuantiFERON-CMV ELISA sample. The left panel indicates qualitative ELISA results stratified by ‘always positive (women 1 – 40)’, ‘always negative (women 41 – 55)’, and ‘mixed’ (women 56 – 61) responses. The middle panel (heatmap) indicates the corresponding quantity of IFNγ released in IU/ml (sorted from high [top of figure] to low [bottom of figure] using first available IFNγ quantity for each woman), with warmer colours (e.g., red) signifying higher values and cooler colours (e.g., green) representing lower values. The right panel indicates HCMV shedding and quantity (in IU/ml) in saliva (S, ▲), urine (U, ●), and vaginal secretions (V, ◆). Blue cells indicate absence of HCMV shedding. White cells in all panels indicate missing samples.

Abbreviations: ELISA, enzyme-linked immunosorbent assay; Indm, indeterminate (due to a mitogen level < 0.5 IU/ml), Neg, negative; Pos, positive; T, timepoint

*Supplementary Figure 9*

Natural history of T-cell immune responses during pregnancy (T1 to T3) and postpartum (T4) as assessed by T-SPOT.CMV ELISPOT for 31 women who had more than one T-SPOT.CMV ELISPOT sample. The left panel indicates qualitative ELISPOT results stratified by ‘always positive (women 1 – 29)’ and ‘mixed (women 30 – 31)’ global responses. The middle panel (heatmap) indicates the corresponding quantity of IFNγ released in spot count (sorted from high (top of figure) to low (bottom of figure) using first available spot count for IE1 antigen for each woman) on stimulation with IE1 and pp65 antigens, with warmer colours (e.g., red) signifying higher values and cooler colours (e.g., green) representing lower values. The right panel indicates HCMV shedding and quantity (in IU/ml) in saliva (S, ▲), urine (U, ●), and vaginal secretions (V, ◆). Blue cells indicate absence of HCMV shedding. White cells in all panels indicate missing samples.

Abbreviations: ELISPOT, enzyme-linked immunospot; IE1, intermediate-early 1 antigen; Neg, negative; Pos, positive; pp65, phosphoprotein 65 antigen; T, timepoint.

*Supplementary Figure 10*

Agreement between QuantiFERON-CMV ELISA and T-SPOT.CMV ELISPOT for detecting T-cell immune responses. QuantiFERON-CMV and T-SPOT.CMV qualitative results that were in agreement are highlighted in grey. Indeterminate QuantiFERON-CMV result is due to a mitogen level of <0.5 IU/ml.

| **Timepoint** | **QuantiFERON-CMV** | **T-SPOT.CMV** | **n** |
| --- | --- | --- | --- |
| 1 | Pos | Pos^2^ | 36 |
|  | Neg | Neg | 0 |
|  | Pos | Neg | 0 |
|  | Neg | Pos^2^ | 12 |
| 2 | Pos | Pos^2^ | 16 |
|  | Neg | Neg | 0 |
|  | Pos | Neg | 1 |
|  | Neg | Pos | 8 |
| 3 | Pos | Pos^3^ | 20 |
|  | Neg | Neg | 1 |
|  | Pos | Neg | 0 |
|  | Neg | Pos | 4 |
|  | Indeterminate | Pos | 1 |
| 4 | Pos | Pos | 4 |
|  | Neg | Neg | 0 |
|  | Pos | Neg | 0 |
|  | Neg | Pos^1^ | 3 |
|  | Indeterminate | Pos | 1 |

Abbreviations: ELISA; enzyme-linked immunosorbent assay; ELISPOT, enzyme-linked immunospot; n, number of samples; neg, negative; pos, positive; T-SPOT.

^1, 2, 3^ Denotes number of sample(s) that had negative IE1 but positive pp65 immune responses.

*Supplementary Figure 11*

Agreement (highlighted in grey) between QuantiFERON-CMV ELISA and T-SPOT.CMV ELISPOT for detecting T-cell immune responses, stratified by shedding status.

| **Shedding** | **Timepoint** | **QFN** | **T-SPOT** | **n** | **In agreement (#/n, % [95% CI])** | **Not in agreement (#/n, % [95% CI])** |
| --- | --- | --- | --- | --- | --- | --- |
| Yes | 1 | Pos | Pos^1^ | 3 | 11/16  68.8%  [44.4, 85.8] | 5/16 |
|  |  | Neg | Neg | 0 |  | 31.2% |
|  |  | Pos | Neg | 0 |  | [14.2, 55.6] |
|  |  | Neg | Pos | 2 |  |  |
|  | 2 | Pos | Pos | 4 |  |  |
|  |  | Neg | Neg | 0 |  |  |
|  |  | Pos | Neg | 1 |  |  |
|  |  | Neg | Pos | 2 |  |  |
|  | 3 | Pos | Pos^1^ | 4 |  |  |
|  |  | Neg | Neg | 0 |  |  |
|  |  | Pos | Neg | 0 |  |  |
|  |  | Neg | Pos | 0 |  |  |
|  | 4 | Pos | Pos | 0 |  |  |
|  |  | Neg | Neg | 0 |  |  |
|  |  | Pos | Neg | 0 |  |  |
|  |  | Neg | Pos | 0 |  |  |
| No | 1 | Pos | Pos^1^ | 33 | 65/90 | 25/90 |
|  |  | Neg | Neg | 0 | 72.2% | 27.8% |
|  |  | Pos | Neg | 0 | [62.2, 80.4] | [19.6, 37.8] |
|  |  | Neg | Pos^2^ | 10 |  |  |
|  | 2 | Pos | Pos^2^ | 11 |  |  |
|  |  | Neg | Neg | 0 |  |  |
|  |  | Pos | Neg | 0 |  |  |
|  |  | Neg | Pos | 6 |  |  |
|  | 3 | Pos | Pos^2^ | 16 |  |  |
|  |  | Neg | Neg | 1 |  |  |
|  |  | Pos | Neg | 0 |  |  |
|  |  | Neg | Pos | 4 |  |  |
|  |  | Indm | Pos | 1 |  |  |
|  | 4 | Pos | Pos | 4* |  |  |
|  |  | Neg | Neg | 0 |  |  |
|  |  | Pos | Neg | 0 |  |  |
|  |  | Neg | Pos^1^ | 3* |  |  |
|  |  | Indm | Pos | 1* |  |  |
| Abbreviations: n, number of samples; QFN, QuantiFERON-CMV; T-SPOT, T-SPOT.CMV.  * One participant had paired urine and saliva swab but declined vaginal swab. | | | | | | |
| ^1, 2^ Denotes number of sample(s) that had negative IE1 but positive pp65 responses. | | | | | | |
|  | | | | | |  |
